# Cluster ensemble based on Random Forests for genetic data

**DOI:** 10.1186/s13040-017-0156-2

**Published:** 2017-12-15

**Authors:** Luluah Alhusain, Alaaeldin M. Hafez

**Affiliations:** College of Computer and Information Sciences, King Saud University, Riyadh, Saudi Arabia

**Keywords:** Cluster ensemble, Random Forests, Genetic population, Population structure analysis, Random Forest proximity, High-dimensional data, Ensemble diversity, Single nucleotide polymorphism, Normalized mutual information

## Abstract

**Background:**

Clustering plays a crucial role in several application domains, such as bioinformatics. In bioinformatics, clustering has been extensively used as an approach for detecting interesting patterns in genetic data. One application is population structure analysis, which aims to group individuals into subpopulations based on shared genetic variations, such as single nucleotide polymorphisms. Advances in DNA sequencing technology have facilitated the obtainment of genetic datasets with exceptional sizes. Genetic data usually contain hundreds of thousands of genetic markers genotyped for thousands of individuals, making an efficient means for handling such data desirable.

**Results:**

Random Forests (RFs) has emerged as an efficient algorithm capable of handling high-dimensional data. RFs provides a proximity measure that can capture different levels of co-occurring relationships between variables. RFs has been widely considered a supervised learning method, although it can be converted into an unsupervised learning method. Therefore, RF-derived proximity measure combined with a clustering technique may be well suited for determining the underlying structure of unlabeled data. This paper proposes, RFcluE, a cluster ensemble approach for determining the underlying structure of genetic data based on RFs. The approach comprises a cluster ensemble framework to combine multiple runs of RF clustering. Experiments were conducted on high-dimensional, real genetic dataset to evaluate the proposed approach. The experiments included an examination of the impact of parameter changes, comparing RFcluE performance against other clustering methods, and an assessment of the relationship between the diversity and quality of the ensemble and its effect on RFcluE performance.

**Conclusions:**

This paper proposes, RFcluE, a cluster ensemble approach based on RF clustering to address the problem of population structure analysis and demonstrate the effectiveness of the approach. The paper also illustrates that applying a cluster ensemble approach, combining multiple RF clusterings, produces more robust and higher-quality results as a consequence of feeding the ensemble with diverse views of high-dimensional genetic data obtained through bagging and random subspace, the two key features of the RF algorithm.

## Background

Clustering is an unsupervised learning technique aimed at uncovering the underlying natural structure of data. In data analysis, clustering is the process of partitioning objects into groups based on their similarities, where objects in the same group are more similar to one another than to objects in different groups. Clustering plays an essential role in several application domains, such as text mining, image segmentation, and bioinformatics. In bioinformatics, clustering has been extensively used as an approach for detecting interesting patterns in genetic data. Such an approach is formally used to find the underlying population substructure from genetic data without considering prior information. The analysis of population structures is a crucial prerequisite for any further analysis of genetic data, such as genome-wide association mapping [1] for reducing false positive rates, and forensics [2] for developing reference panels to provide information on an individual’s ancestry. This kind of analysis aims to group individuals into subpopulations based on shared genetic variations. Single nucleotide polymorphisms (SNPs) are the most common type of genetic variation used to infer population structure. SNPs occur when a single nucleotide from a DNA sequence differs at the same position between individuals. An SNP has three categories: homozygous with the common allele (genotype AA), heterozygous (genotype AB), and homozygous with the rare allele (genotype BB). Advances in DNA sequencing technology have facilitated the attainment of genetic datasets with exceptional sizes. Genetic data usually contain hundreds of thousands of genetic markers genotyped for thousands of individuals. Thus, an efficient means for handling such high-dimensional data is desirable.

Two major clustering approaches have been developed to infer the structure of populations from genetic data: distance-based and dimension reduction-based approaches. AWclust [3] is a distance-based approach that consists of constructing an allele-sharing distance (ASD) matrix between all pairs of individuals in the genetic data. It then applies hierarchical clustering to infer clusters of individuals from the ASD matrix using Ward’s algorithm. PCAclust [4] is a dimension reduction-based clustering approach that involves applying principal component analysis (PCA) to reduce the dimensions of the genetic data. It then applies a model-based clustering algorithm (i.e., a Gaussian mixture model clustering) to the set of relevant principal components.

Inferring population structures from genetic data can be defined as a problem of determining how to assign *N* individuals using *l* genetic markers to *K* subpopulations. This paper proposes a new approach for inferring population structures from genetic data. The proposed approach is based on Random Forests (RFs). Our motivation for using RFs is twofold: First, its capability of handling high-dimensional data of thousands of individuals and hundreds of thousands of markers, which makes it a suitable solution for the problem of population structure analysis. Second, RFs provides a natural method for measuring proximities between individuals; this measure weighs the co-occurrence between markers such that the more correlated a marker is with other markers, the more it will affect the proximity between individuals. Therefore, it can handle the linkage nature among genetic markers. In genetics, *linkage* refers to a correlation between types of an allele that appear at different loci, especially when a genome is densely genotyped due to linkage disequilibrium (LD) [5]. LD refers to the non-random association of particular alleles, which plays a major role in discovering population structures from genetic data. RF clustering, in which RF-derived proximity is combined with a clustering technique, is well suited for discovering the underlying structure of unlabeled data [6, 7]. However, the main concern underlying the RF algorithm is that, for each run, a different proximity matrix is generated due to its random nature, therefore producing a different clustering result each time. Thus, this paper proposes a Random Forest cluster Ensemble (RFcluE) approach to discover the underlying structure of genetic data. Within this approach, a cluster ensemble framework is utilized to combine the results of multiple runs of RF clustering toward obtaining a more reliable and robust clustering result than a single run of RF clustering.

## Methods

### Random Forests

Random Forests (RFs) has emerged as an efficient algorithm capable of handling high-dimensional data [8]. RFs was formally developed by Leo Breiman [8] as a classification and regression ensemble learning method. This method is based on a combination of bagging [9] and random subspace [10]. *Bagging* is the process of aggregating the results of multiple trees, where each tree is grown on a bootstrap sample of the objects. A bootstrap sample of a specified size is drawn with replacement from the original data. *Random subspace* refers to the selection of a random subset of variables as candidates for splitting at each node. Rather than considering all variables as candidates for splitting, RFs considers only a subset of variables, thus reducing the correlation between trees.

In the context of population structure analysis, individuals are the objects, while SNP markers are the variables. Thus, in RFs, a forest is constructed by building multiple decision trees. To build a tree, the algorithm first creates a root node containing a bootstrap sample of the individuals. Then, at each node, the algorithm selects a random subset of the markers to search over, and subsequently determines the best split markers based on a splitting criterion. A splitting criterion usually maximizes some measure of node purity, which means the degree to which individuals of a node belong to one class. In RFs, the Gini index [11] is used as a splitting criterion to select the best split at each node. The Gini index measures how well a potential split of a node is in separating the individuals into two known classes. Consequently, the Gini index at node *n* is defined as:

1 $$ Gini(n)=\sum \limits_{c=1}^C{\overset{\hat{\mkern6mu} }{p}}_c^n\left(1\kern0.5em -\kern0.5em {\overset{\hat{\mkern6mu} }{p}}_c^n\right) $$

where ${\overset{\hat{\mkern6mu} }{p}}_c^n=\frac{n_c}{n}$ is the proportion of individuals that are of class *c* at node *n*. The Gini index is minimized when all individuals in the node are of the same class, increasing as the individuals in the node are spread more evenly among different classes. The gain for splitting node *n* based on marker *x*_*i*_, *Gain* (*x*_*i*_, *n*), is defined as the difference between the impurity at node *n* and the weighted average of impurities at each child node of *n*. That is,

2 $$ Gain\ \left({x}_i,n\right)= Gini\left({x}_i,n\right)-{w}_L Gini\left({x}_i,{n}^L\right)-{w}_R Gini\left({x}_i,{n}^R\right) $$

where *n* ^*L*^ and *n* ^*R*^ are the left and right child nodes of the parent node *n*, respectively, and *w*_*L*_ and *w*_*R*_ are the proportions of individuals assigned to the left and right child nodes. Based on the gain value, the marker *x*_*i*_ with the lowest impurity is selected to split individuals at node *n*.

This process of splitting is repeated until an unpruned tree is formed. The generated forest contains a significant amount of information about the relationship between the markers and the individuals that can be used for prediction, variable importance, proximity calculation, missing data imputation, and outlier detection. RF-derived proximity, a byproduct of a random forest, is defined based on similar individuals ending up in the same leaf node more often than dissimilar individuals. This proximity can capture different levels of co-occurring relationships between markers.

RFs is widely considered a supervised learning method, although it can be adapted as an unsupervised learning method to derive proximity matrix from unlabeled data [6]. Recently, unsupervised RFs has been successfully applied in a wide variety of domains, including bioinformatics [6, 12], image and document analysis [13–15], networking [16], cloud computing [17, 18], manufacturing [19], remote sensing [20], and chemometrics [21].

To use RFs for unsupervised learning, the RF algorithm must first randomly generate synthetic data based on the original dataset, in which a random forest is built to distinguish the original data from the synthetic data. One approach for generating the synthetic data is to randomly draw synthetic individuals from marginal distributions of each observed marker in the original data [4]. Hence, the synthetic class has a distribution of independent random markers, where each marker follows the same distribution as the corresponding marker in the original data.

### Cluster ensemble

A cluster ensemble is an effective approach for combining different clusterings of the same dataset into a more robust and higher-quality clustering than any individual clustering. A cluster ensemble typically consists of two components: an ensemble constructor and a consensus function. An ensemble constructor generates a set of different partitions of the dataset, which is referred to as “base clusterings” or “ensemble members.” On the other hand, a consensus function combines the base clusterings of the ensemble and produces a single clustering as the ultimate output of the cluster ensemble.

Regarding the ensemble constructor, several methods have been proposed to obtain ensemble members, including applying different clustering algorithms [22, 23], applying the same clustering algorithm with random parameter initializations [24–26], projecting data onto different subspaces [26–28], and data subsampling [25, 29, 30].

The consensus function is critical in the cluster ensemble for performing the combination task. Different approaches have been proposed, including feature-based, graph-based, and pairwise-based approaches. The feature-based approach deals with the problem of cluster ensemble as the clustering of categorical data [31, 32]. Specifically, each ensemble member provides a cluster label as a new feature describing each object. Thus, any categorical clustering can be exploited to find the consensus clustering. The graph-based approach represents the cluster ensemble as a graph, which is then divided into a number of clusters using graph partition technique [26, 28]. Lastly, the pairwise-based approach represents the information from multiple base clusterings as a co-association matrix that contains co-occurrence relationships between all pairs of objects, which can be used as an input to any similarity-based clustering to derive the final partition [23, 25, 27, 33, 34].

### Cluster ensemble based on Random Forests

**Fig. 1:**
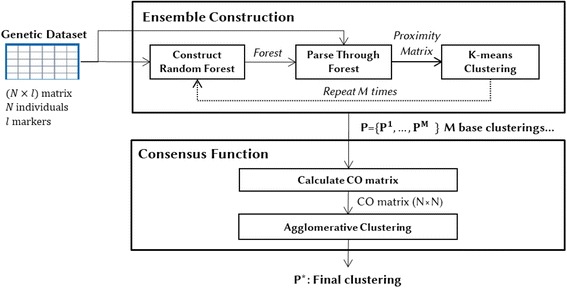
Random Forest cluster Ensemble (RFcluE) approach

The proposed approach, the Random Forest cluster Ensemble (RFcluE), is based on the concept of a cluster ensemble, where RF clustering is used as a base clustering method. The general framework for the RFcluE approach is shown in Fig. 1. The RFcluE approach has two stages: The first stage is ensemble construction, followed by the consensus function stage. The first stage takes a genetic dataset as an input and then outputs a set of partitions. The second stage takes the set of partitions as an input and produces a final clustering result as an output.

Let *G* = {*g*_1_, *g*_2_, …, *g*_*N*_} represent a set of *N* individuals, where *g*_*i*_ is a genotype profile of individual *i* that consists of *l* genetic markers. A cluster ensemble first constructs a set of partitions (i.e., ensemble members), *P* = {*P*_1_, *P*_2_, …, *P*_*M*_}, by applying the base clustering method *M* times. Each run of the base clustering method returns a set of clusters,${P}_i=\left\{{C}_i^1,{C}_i^2,\dots .,{C}_i^{k_i}\right\},$such that ${\bigcup}_{j=1}^{k_i}{C}_i^j=G$, where *k*_*i*_ is the number of clusters in the *i* ^th^ clustering and ${C}_i^j$ is the *j* ^th^ cluster of the *i* ^th^ partition, for *i* = 1, 2, …, *M*. Then, the consensus function is applied to the set of generated partitions, *P*, in order to find a new partition, *P* ^∗^, that better represents the properties of each partition in *P* of the cluster ensemble.

#### Ensemble construction

The ensemble construction based on RFs is used to create the base clusterings. Clustering using RFs is generally composed of three steps:

(i) Constructing a forest in an unsupervised fashion.
(ii) Parsing the constructed forest to compute the proximities between individuals.
(iii) Applying a clustering technique on the resulting proximity matrix.

The input of the ensemble constructor is a genetic dataset, *G ЄR* ^*N* × *l*^, where *N* is the number of individuals and *l* is the number of genetic markers; and four parameters, specifically the number of trees (*ntrees*), the tree size controlled by specifying the maximum number of leaf nodes (*MN*), the number of clusters in each partition (*k*), and the ensemble size (*M*).

Since the base clustering method of the ensemble is RF clustering, the ensemble constructor first computes the RF-derived proximity matrix. The algorithm that builds a random forest, *RF*, of size *ntrees* trees, where each tree has a maximum of *MN* leaf nodes in the unsupervised mode, is described in Algorithm 1. Based on the constructed forest, the RF-derived proximity matrix, which denotes the similarity between each pair of individuals of size *N* × *N*, is calculated. Then, the proximity matrix *S* is converted to a dissimilarity matrix, *D*, by using $D=\sqrt{\left(1-s\right)}$. Lastly, the method applies K-means on this dissimilarity, after transforming it to Euclidean space using multidimensional scaling (MDS) [35], to partition the individuals into *k* clusters. The MDS technique used is classical scaling, where a *N* × *N* distance matrix is converted into a *N* × *p* configuration matrix. The configuration matrix contains the coordinates of *N* individuals in *p*-dimensional space, where *p* < *N*; *p* is determined such that the dimension of the smallest space in which *N* individuals can be embedded, given *D* that contains the inter-distances between individuals.

The output of the base clustering, RF clustering, is a single partition of the data. To construct a cluster ensemble of size *M* partitions, the base clustering method is repeated *M* times and, for each run, a different partition of data is generated such that the cluster ensemble is *P* = {*P*_1_, *P*_2_, …, *P*_*M*_}. The pseudo-code of the ensemble construction of RFcluE is outlined in Algorithm 2.
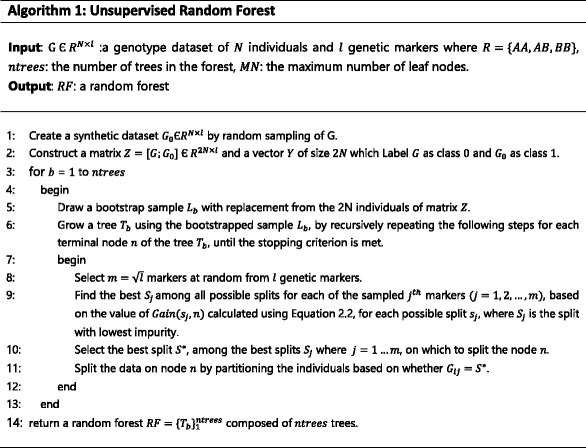
 
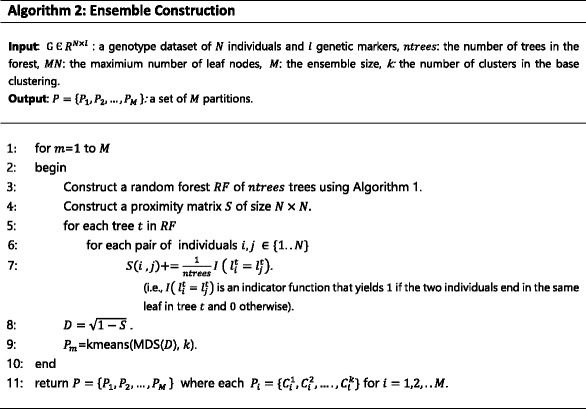


#### Consensus function

Given a cluster ensemble *P*, *P* contains a set of *M* partitions,*P* = {*P*_1_, *P*_2_, …, *P*_*M*_}, produced by the ensemble construction. Each partition *P*_*i*_ returns a set of clusters such that ${P}_i=\left\{{C}_i^1,{C}_i^2,\dots .,{C}_i^{k_i}\right\}$, where *k*_*i*_ is the number of clusters in *P*_*i*._ Each partition *P*_*i*_ contains the cluster labels of *N* individuals, such that *c*(*n*) denotes the cluster label to which the individual *n* belongs. The goal of the consensus function is to find a new partition, *P* ^∗^,that combines the information from the cluster ensemble *P*. The pseudo-code of the consensus function of RFcluE is outlined in Algorithm 3. It works as follows. First, the consensus function calculates the co-association matrix (CO). CO summarizes the information in the ensemble *P* as the *N* × *N* matrix. This matrix denotes the similarity between any pair of *N* individuals as a proportion of *M* partitions in the ensemble *P*, in which they are assigned to the same cluster. Then, the consensus function applies agglomerative hierarchical clustering based on Ward’s minimum variance algorithm [36, 37] on the CO matrix to obtain the final partition, *P* ^∗^. Ward’s algorithm is utilized because the inference of population structure needs an algorithm that minimizes the increase of within-cluster variance each time an individual is added to a cluster.
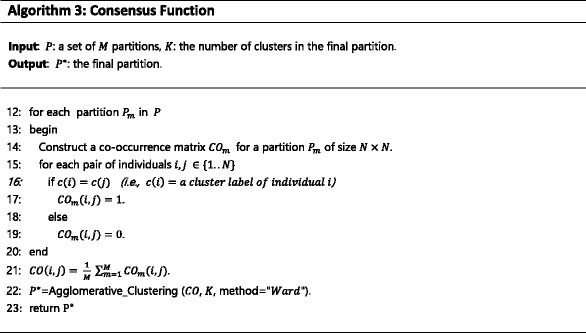


### Datasets

The performance of the RFcluE approach was empirically evaluated on three well-known real datasets, namely a human genotype dataset from the Pan-Asian database [38], worldwide human genotype data from the HapMap project [39], and the worldwide human SNP dataset provided by Prof. Mark D. Shriver and described in [40, 41]. The population (i.e., truth label) that an individual belongs to is known for all individuals in all datasets. Table 1 describes the used datasets in terms of the number of individuals, the number of SNPs, and the number of populations.

**Table 1:** The description of real genetic datasets

Dataset	Number of Individuals	Number of SNPs	Number of Populations
HapMap	762	46,256	11
Pan-Asian	443	54,794	10
Shriver’s	274	10,805	12

### Evaluation metrics

Many experiments were conducted to investigate the performance of the RFcluE approach. The performance evaluation comprised an assessment of the quality of the final clustering result of the approach. Besides, an assessment of the quality and diversity of the base clusterings, which are generated by the ensemble constructor, was conducted in order to study their impact on performance. Both quality and diversity were evaluated based on normalized mutual information (NMI).

NMI is a measure of agreement between two partitions based on information theory [28]. It treats the two partitions as nominal random variables. The NMI score between two partitions, *A* and *B*, is computed as:

3 $$ \mathrm{NMI}\left(\mathrm{A},\mathrm{B}\right)=\frac{\mathrm{MI}\left(\mathrm{A},\mathrm{B}\right)}{\left(\mathrm{H}\left(\mathrm{A}\right)+\mathrm{H}\left(\mathrm{B}\right)\right)/2} $$

*MI*(*A*, *B*) is the mutual information between two partitions, *A* and *B*, calculated as follows:

4 $$ \mathrm{MI}\left(\mathrm{A},\mathrm{B}\right)=\sum \limits_{\mathrm{i}=1}^{{\mathrm{k}}_{\mathrm{A}}}\sum \limits_{\mathrm{j}=1}^{{\mathrm{k}}_{\mathrm{B}}}\frac{{\mathrm{N}}_{\mathrm{i}\mathrm{j}}}{\mathrm{N}}\ \log \left(\frac{{\mathrm{N}}_{\mathrm{i}\mathrm{j}}\ \mathrm{N}}{{\mathrm{N}}_{\mathrm{i}.}\ {\mathrm{N}}_{.\mathrm{j}}}\right) $$

*H*(*A*) and *H*(*B*) are the entropy of partition *A* and partition *B*, respectively, and are calculated as:

5 $$ \mathrm{H}\left(\mathrm{A}\right)=-\sum \limits_{\mathrm{i}=1}^{{\mathrm{k}}_{\mathrm{A}}}\frac{{\mathrm{N}}_{\mathrm{i}.}}{\mathrm{N}}\ \log \left(\frac{{\mathrm{N}}_{\mathrm{i}}.}{\mathrm{N}\ }\right) $$

6 $$ \mathrm{H}\left(\mathrm{B}\right)=-\sum \limits_{\mathrm{j}=1}^{{\mathrm{k}}_{\mathrm{B}}}\frac{\mathrm{N}{.}_{\mathrm{j}}}{\mathrm{N}}\ \log \left(\frac{\mathrm{N}{.}_{\mathrm{j}}}{\mathrm{N}\ }\right) $$

where *k*_*A*_ is the number of clusters in partition *A*, *k*_*B*_ is the number of clusters in partition *B*, *N*_*i*_ is the number of individuals in cluster *i* (*C*_*i*_) of partition *A*, *N*_*j*_ is the number of individuals in cluster *j* (*C*_*j*_) of partition *B*, and *N*_*ij*_ is the number of shared individuals between cluster *i* of partition *A* and cluster *j* of partition *B* (*C*_*i*_ ∈ *A* and *C*_*j*_ ∈ *B*).

Therefore, the NMI score becomes:

7 $$ \mathrm{NMI}\left(\mathrm{A},\mathrm{B}\right)=\frac{-2\ {\sum}_{\mathrm{i}=1}^{{\mathrm{k}}_{\mathrm{A}}}{\sum}_{\mathrm{j}=1}^{{\mathrm{k}}_{\mathrm{B}}}{\mathrm{N}}_{\mathrm{i}\mathrm{j}}\ \log \left(\frac{{\mathrm{N}}_{\mathrm{i}\mathrm{j}}\ \mathrm{N}}{{\mathrm{N}}_{\mathrm{i}.}\ {\mathrm{N}}_{.\mathrm{j}}}\right)}{\sum_{\mathrm{i}=1}^{{\mathrm{k}}_{\mathrm{A}}}{\mathrm{N}}_{\mathrm{i}.}\ \log \left(\frac{{\mathrm{N}}_{\mathrm{i}.}}{\mathrm{N}\ }\right)+{\sum}_{\mathrm{j}=1}^{{\mathrm{k}}_{\mathrm{B}}}{\mathrm{N}}_{.\mathrm{j}}\ \log \left(\frac{{\mathrm{N}}_{.\mathrm{j}}}{\mathrm{N}\ }\right)} $$

Note that 0 ≤ *NMI* (*A*, *B*) ≤ 1 , so it takes its maximum value if partitions *A* and *B* are identical, and its minimum value if partitions *A* and *B* are independent.

Let *P* represent a cluster ensemble that contains a set of generated *M* base partitions *P* = {*P*_1_, *P*_2_, …, *P*_*M*_}, *P* ^∗^ is the final clustering result of the cluster ensemble approach, and *L* is the truth population labels of individuals.

Based on NMI, the quality of the final clustering result *P* ^∗^ of an ensemble *P* is calculated as follows:

8 $$ \mathrm{Q}\left({\mathrm{P}}^{\ast}\right)=\mathrm{NMI}\left({\mathrm{P}}^{\ast },\mathrm{L}\right) $$

The diversity between two partitions, *P*_*i*_, *P*_*j*_,is denoted as (1 − *NMI*(*P*_*i*_, *P*_*j*_ ) ). Therefore, the diversity of an ensemble *P* is the average of all pairwise diversities among all pairs of partitions—*P*_*i*_, *P*_*j*_ ∈ *P*—and can be calculated as follows:

9 $$ \mathrm{DS}\left(\mathrm{P}\right)=\frac{2}{\mathrm{M}\ \left(\mathrm{M}-1\right)}\sum \limits_{\mathrm{i}=1}^{\mathrm{M}-1}\sum \limits_{\mathrm{j}=\mathrm{i}+1}^{\mathrm{M}}\left(1-\mathrm{NMI}\left({\mathrm{P}}_{\mathrm{i}},\kern0.5em {\mathrm{P}}_{\mathrm{j}}\ \right)\right) $$

where the higher the *DS*(*P*) value, the more diverse the ensemble.

The quality of cluster ensemble *P* is the average quality of all partitions, *P*_*i*_ ∈ *P*, and can be calculated as follows:

10 $$ \mathrm{Q}\left(\mathrm{P}\right)=\frac{1}{\mathrm{M}\ }\ \sum \limits_{\mathrm{i}=1}^{\mathrm{M}}\mathrm{NMI}\left({\mathrm{P}}_{\mathrm{i}},\mathrm{L}\ \right) $$

In the comparison study, the adjusted Rand index (ARI) and accuracy (AC) were used, in addition to NMI.

The ARI [42] is a variation of the Rand index [43] that measures how often similar individuals are assigned to the same cluster and dissimilar individuals to different clusters. Given two partitions, *A* and *B*, the ARI between A and B is calculated as follows:

11 $$ \mathrm{ARI}\left(\mathrm{A},\mathrm{B}\right)=\frac{\sum_{\mathrm{i}=1}^{{\mathrm{k}}_{\mathrm{A}}}{\sum}_{\mathrm{j}=1}^{{\mathrm{k}}_{\mathrm{B}}}\left(\begin{array}{c}{\mathrm{N}}_{\mathrm{i}\mathrm{j}}\\ {}2\end{array}\right)-\frac{2\ {\sum}_{i=1}^{k_A}\left(\begin{array}{c}{N}_{i.}\\ {}2\end{array}\right){\sum}_{j=1}^{k_B}\left(\begin{array}{c}{N}_{.j}\\ {}2\end{array}\right)}{N\left(N-1\right)}}{\frac{1}{2}\ \left({\sum}_{i=1}^{k_A}\left(\begin{array}{c}{N}_{i.}\\ {}2\end{array}\right)+{\sum}_{j=1}^{k_B}\left(\begin{array}{c}{N}_{.j}\\ {}2\end{array}\right)\right)-\frac{2\ {\sum}_{i=1}^{k_A}\left(\begin{array}{c}{N}_{i.}\\ {}2\end{array}\right){\sum}_{j=1}^{k_B}\left(\begin{array}{c}{N}_{.j}\\ {}2\end{array}\right)}{N\left(N-1\right)}} $$

where *k*_*A*_ and *k*_*B*_ are the number of clusters in *A* and *B*, repectively. *N*_*ij*_ is the number of individuals in both cluster *i* of partition *A* and cluster *j* in partition *B*; *N*_*i*._ is the number of individuals in cluster *i* of partition A; and *N*_.*j*_ is the number of individuals in cluster *j* in partition *B*. Obtaining a higher value of ARI is better, while random partitions yield values close to zero.

AC is used to measure the purity of the resulting clusters. To compute AC, each cluster is first assigned to the population label that is most frequent in that cluster. Then, AC is computed by counting the number of correctly assigned individuals and dividing the sum by the total number of individuals, *N*, as follows:

12 $$ \mathrm{AC}=\sum \limits_{\mathrm{i}=1}^{\mathrm{k}}\frac{\left({\mathrm{n}}_{\mathrm{i}}-{\mathrm{m}}_{\mathrm{i}}\right)}{\mathrm{N}} $$

where *N* is the number of individuals, *k* is the number of clusters, *n*_*i*_ is the number of individuals in cluster *i*, and *m*_*i*_ is the number of individuals with the majority population label in cluster *i*.

Since each run of ensemble clustering would generate different results, all the metrics are reported as an average value of 20 random runs.

## Results and discussion

Many experiments were conducted on the real genetic datasets described previously to assess the RFcluE approach in clustering high-dimensional genetic data to infer population structure, including parameter analysis, consensus function, comparison study, and diversity and quality analysis.

### Parameter analysis

The objective of the parameter analysis was to study the impact of the change in the parameters on RFcluE performance. In this analysis, both the diversity and quality of the ensemble (i.e., base clusterings), in addition to the quality of the final clustering, were considered. Running RFcluE involves the choice of two RF parameters, the number of trees in the forest (*ntrees*), and the tree size by specifying the maximum number of leaf nodes (*MN*). In addition to RF parameters, there is the ensemble size *M*, which is the number of times the base clustering method is executed. The last parameter is the number of clusters, *k*, as an input to the base clustering method. For the consensus function, the only parameter to be specified is the number of clusters for the final clustering result. To eliminate its effect in evaluation, the consensus function is forced to divide the individuals into *K* clusters, where *K* is the number of the true populations for the examined datasets. Therefore, the final clustering result can be evaluated against the corresponding truth population labels for the dataset.

**Fig. 2:**
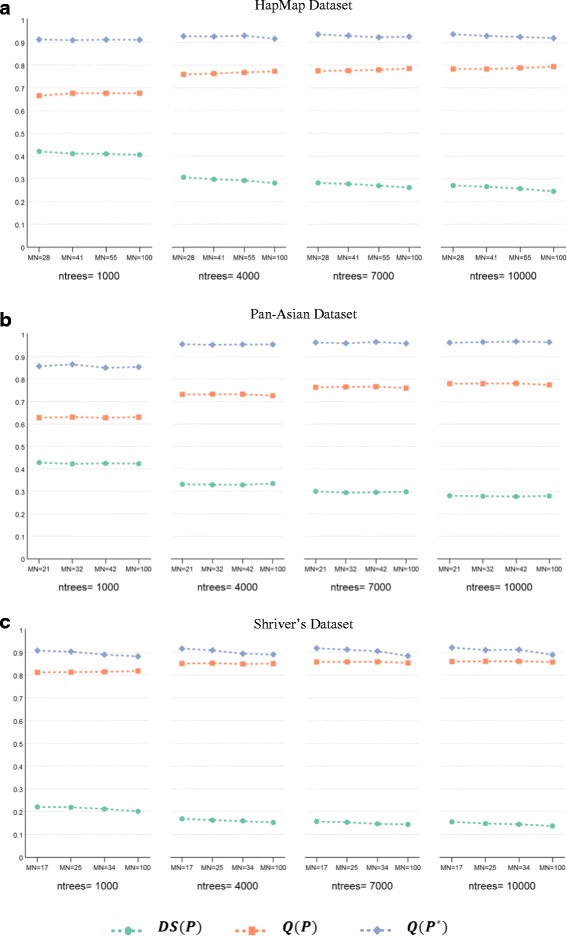
The impact of the change of RF parameters on the performance of the RFcluE approach. The figure shows the impact of the number of trees (*ntrees*) and the tree size controlled by the maximum number of leaf nodes (*MN*) on the performance of the RFcluE approach measured using the diversity and quality of the base clusterings along with the quality of the ensemble’s final clustering, where M = 40. **a** HapMap Dataset. **b** Pan-Asian Dataset. **c** Shriver’s Dataset

Figure 2 plots the values of the diversity and quality of the ensemble as well as the quality of the ensemble’s final clustering to show the impact of the change in RF parameters. For each dataset, we tested these values (*ntrees* = {1000, 4000, 7000, 10000}, $MN=\left\{\sqrt{N,}\ \frac{3}{2}\sqrt{N},2\sqrt{N},100\ \right\}$, *M* = 40, and $k=\sqrt{N}$), where *N* is the number of individuals in the examined dataset. From the plots, we were able to observe the insignificant impact of tree size on the quality of the ensemble’s final clustering of Pan-Asian and HapMap datasets. For Shriver’s dataset, the *MN* parameter had a minor impact, with lower values performing better than higher values. Consequently, we can conclude that the smallest value of the maximum number of leaf nodes, $MN=\sqrt{N}$, is empirically sufficient to control the tree size in the forest. This value is also more efficient as it takes less time to run the RF algorithm. Additionally, the plots show that an increase in the number of trees is associated with a decrease in the diversity and an increase in the quality of the base clusterings, as well as an increase in the quality of the ensemble’s final clustering. These trends varied for each dataset. For the Pan-Asian dataset, there was a positive correlation between the performance improvement of the ensemble clustering and the number of trees, where a significant improvement was seen when the number of trees increased from 1000 to 4000. For the HapMap dataset, we observed similar, albeit minor, improvements as the number of trees increased. For Shriver’s dataset, the performance gain was negligible as the number of trees increased. From these observations, we can conclude that the number of trees is dataset-dependent and must be sufficient to uncover the structure of the examined dataset.

**Fig. 3:**
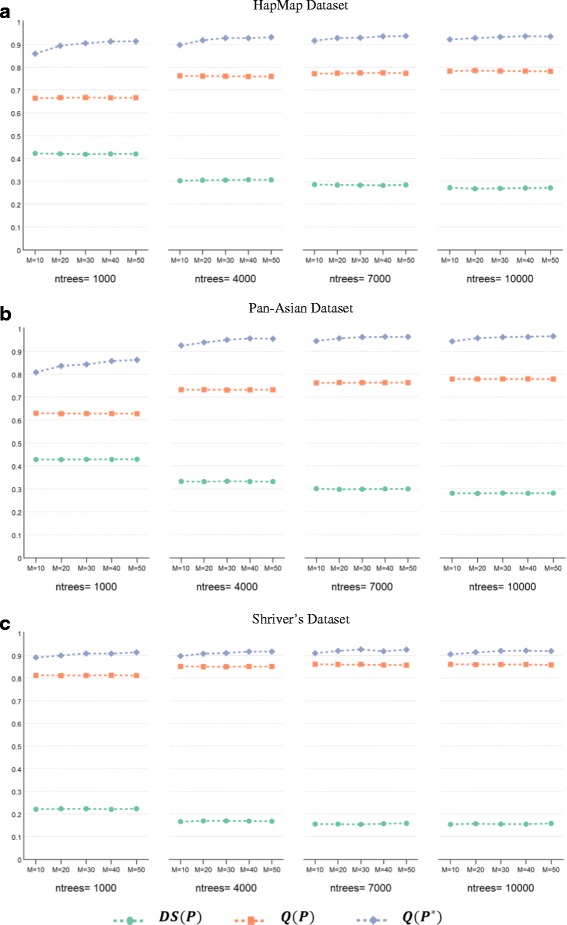
The impact of ensemble size on the performance of the RFcluE approach. The figure shows the impact of the ensemble size (*M*) on the performance of the RFcluE approach, across a different number of trees(*ntrees*), measured using the diversity and quality of the base clusterings along with the quality of the ensemble’s final clustering, where *MN* =$\sqrt{N}$. **a** HapMap Dataset. **b** Pan-Asian Dataset. **c** Shriver’s Dataset

Figure 3 shows the impact of ensemble size on the performance of ensemble clustering, considering both ensemble size and the number of trees. In this figure, the plots report the values of the diversity and quality of the ensemble as well as the quality of the final clustering, where the parameters are: (*M* = {10, 20, 30, 40, 50}, *ntrees* = {1000, 4000, 7000, 10000},$MN=\sqrt{N}$, and $k=\sqrt{N}$). In general, we can see that the diversity and quality of the ensemble are similar across the five different ensemble sizes for all datasets. However, the quality of the ensemble’s final clustering improves as the ensemble size increases. The improvement in overall performance is dependent on the examined dataset, with the Pan-Asian dataset demonstrating the most significant improvement. We can also see that the impact of the ensemble size parameter is diminished as the number of trees in the forest is increased. On the other hand, for Shriver’s dataset, we can see stable performance despite a change in the number of trees and only slight improvement when increasing the ensemble size.

**Fig. 4:**
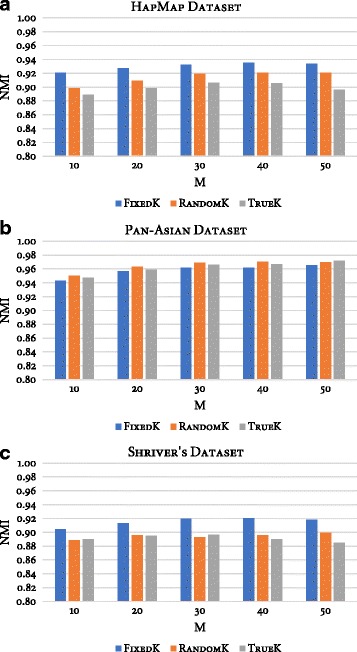
Performance of three schemes for selecting the number of clusters produced by the base clustering method of RFcluE. The figure shows a plot that compares the performance of three schemes—FixedK, RandomK, and TrueK—for selecting the number of clusters produced by the base clustering method in the RFcluE approach over different ensemble sizes, where *ntrees* = 10,000 and *MN* = $\sqrt{N}$. **a** HapMap Dataset. **b** Pan-Asian Dataset. **c** Shriver’s Dataset

The last parameter is the number of clusters, *k*, as an input to the base clustering method. In order to study the impact of this parameter, three schemes were defined to determine the number of clusters, namely *TrueK*, *FixedK*, and *RandomK*. Specifically, let *K* and *N* represent the number of true clusters and the number of individuals in the examined dataset, respectively. The number of clusters for *TrueK* is *k* = *K*; for *FixedK,* the number of clusters is $k=\sqrt{N}$, while for *RandomK* the number is random, selected such that $k\ \epsilon\ \left[2,\sqrt{N}\right]$ for each run of the base clustering method. To compare the performance of the three schemes, an experiment was conducted using these parameters (*M* ={10, 20, 30, 40, 50}, *ntrees* = 10000, $MN=\sqrt{N\ }$). Fig. 4 shows, for each dataset, a bar plot of the NMI values of the three schemes across five ensemble sizes. Regardless of ensemble size, the *FixedK* scheme had higher NMI than the other two schemes for the HapMap and Shriver datasets. As for the Pan-Asian dataset, no significant difference was observed between the three schemes. This observation thus confirms the performance gain of the ensemble’s final clustering when the number of clusters in base clusterings is overproduced. Likewise, this observation also supports the recommendation that the value of *k* be set to greater than the expected number of clusters [44–46].

### Consensus function

**Fig. 5:**
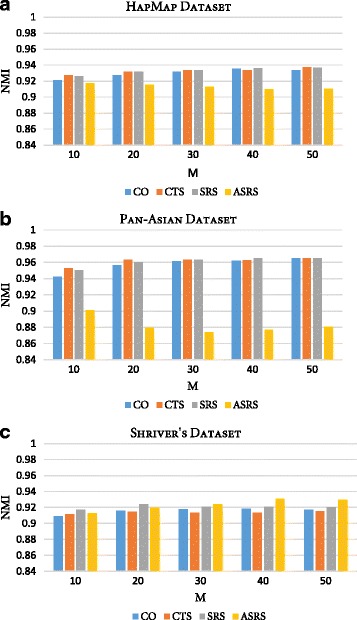
The impact of utilizing different association measures in the consensus function on the performance of the RFcluE. The figures show the NMI of RFcluE when the similarity between partitions is measured using CO, CTS, SRS, and ASRS in the consensus function. **a** HapMap Dataset. **b** Pan-Asian Dataset. **c** Shriver’s Dataset

The consensus function of RFcluE, as presented previously, is composed of calculating the co-association matrix and then applying Ward’s agglomerative clustering. This consensus function performs effectively by exploiting the co-association between individuals in the ensemble. However, the ensemble can be explored by considering the association between clusters within different partitions in addition to the association between individuals. Link-based similarity measures were proposed in [47] to improve the performance of CO by considering the association between clusters. These measures include connected triple-based similarity (CTS), SimRank-based similarity (SRS) and, finally, the approximate SimRank-based similarity (ASRS), which was introduced as an efficient variation of the SRS. Therefore, an experiment was conducted to study the impact of these measures on RFcluE performance when utilizing those measures in the consensus function instead of CO. Fig. 5 shows the NMI of applying CO, CTS, SRS, and ASRS to measure the similarity between different partitions of data in the consensus function. The consensus function was applied to the same ensemble, which was generated using this parameter settings (*ntrees* = 10000,$MN=\sqrt{N}$, and $k=\sqrt{N}$). For all datasets, CO, CTS, and SRS demonstrated comparable performance, while ASRS provided the worst performance compared with other measures for the Pan-Asian dataset. However, ASRS provided the best performance for Shriver’s dataset when the ensemble size was greater than 30. However, the difference in performance between these measures was not statistically significant, with a *p*-value < 0.05.

**Fig. 6:**
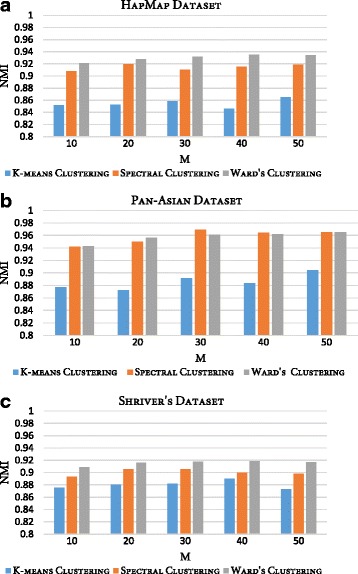
The impact of utilizing different clustering techniques in the consensus function on the performance of the RFcluE. The figure shows the NMI of the RFcluE when applying K-means, spectral clustering, and Ward’s algorithm in the consensus function. **a** HapMap Dataset. **b** Pan-Asian Dataset. **c** Shriver’s Dataset

CO used in the consensus function of RFcluE, represents a similarity matrix in which any similarity-based clustering can be applied to obtain the final clustering result. In RFcluE, we applied Ward’s agglomerative hierarchical clustering. However, different clustering techniques can be applied to the CO, such as K-means and spectral clustering. Therefore, another experiment was conducted wherein these clustering techniques were applied to the CO to examine their impact on the performance of RFcluE. Fig. 6 shows the NMI of the three clustering techniques—Ward’s, K*-*means, and spectral clustering—when applied to the same ensemble. The parameter settings used for the base clustering method were (*M* = {10, 20, 30, 40, 50}, *ntrees* = 10000,$MN=\sqrt{N}$, and $k=\sqrt{N}$). We can see that Ward’s clustering, applied with RFcluE, has the best performance compared with K-means and spectral clustering across all the examined datasets, demonstrating statistically significant performance, with a *p*-value < 0.05.

### Comparison study

**Fig. 7:**
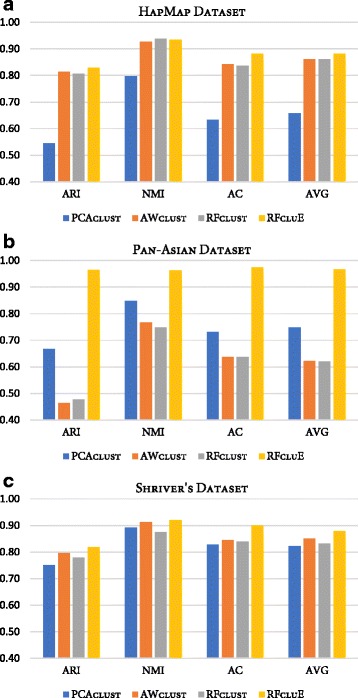
Performance of PCAclust, AWclust, RFclust, and RFcluE evaluated using ARI, AC, and NMI. The figure shows a plot that compares the performance of PCAclust, AWclust, RFclust, and RFcluE, measured using three measures—ARI, AC, and NMI—along with the average of these measures. **a** HapMap Dataset. **b** Pan-Asian Dataset. **c** Shriver’s Dataset

A comparison study was conducted to assess the performance of the proposed approach, RFcluE, against AWclust [48] and PCAclust [4], the two most popular methods for population structure analysis. Moreover, the performance of the RFcluE approach was compared against RFclust. Table 2 and Fig. 7 present the performance of PCAclust, AWclust, RFclust, and RFcluE on the real datasets evaluated using ARI, AC, and NMI. In RFcluE, the clustering result is based on combining multiple runs of RF clustering using a cluster ensemble framework, while RFclust is a clustering method that calculates the average of proximities derived from multiple runs of the RF algorithm and then applies Ward’s agglomerative hierarchical clustering. For RFcluE, the ensemble size *M* = 40 and *FixedK* scheme were used. For RFclust, the number of forests was equal to the ensemble size. For both RFcluE and RFclust, the RF parameters were set such that *ntrees* = 10000 and $MN=\sqrt{N}$. All the compared methods were forced to divide the data into the real number of clusters in the examined dataset. Below, a discussion of the performance of RFcluE, AWclust, and PCAclust is presented, followed by a detailed comparison between RFcluE and RFclust under the same RF parameter settings.

**Table 2:** A performance comparison between PCAclust, AWclust, RFclust, and RFcluE

Dataset	Measure	Methods			
		PCAclust	AWclust	RFclust	RFcluE
HapMap	ARI	0.5453	0.8135	0.8065	0.8282
	NMI	0.7963	0.9277	0.9388	0.9353
	AC	0.6326	0.8412	0.8365	0.882
	AVG	0.6581	0.8608	0.8606	0.8818
Pan-Asian	ARI	0.6668	0.4631	0.4766	0.9644
	NMI	0.8483	0.7663	0.749	0.962
	AC	0.7314	0.6366	0.6363	0.9745
	AVG	0.7488	0.622	0.6206	0.9669
Shriver’s	ARI	0.7502	0.7952	0.7795	0.8184
	NMI	0.8914	0.9121	0.8758	0.9204
	AC	0.8267	0.8448	0.8388	0.8989
	AVG	0.8228	0.8507	0.8314	0.8792

The table shows the performance of PCAclust, AWclust, RFclust, and RFcluE across the real datasets evaluated using ARI, AC, and NMI, along with an average of these three measures (AVG)

#### RFcluE, AWclust, and PCAclust

The performance of the RFcluE, AWclust, and PCAclust approaches, based on ARI, AC, and NMI measures, on three real datasets is compared in Table 2. Fig. 7 shows that RFcluE generally outperforms PCAclust and AWclust over the three datasets. The bar plot for the Pan-Asian dataset indicates that RFcluE yields a superior clustering result when compared to the other approaches based on ARI, AC, and NMI. For the HapMap dataset, PCAclust had the worst performance, while RFcluE had the best performance. For Shriver’s dataset, all approaches had comparable performance, while RFcluE performed better than the other approaches considering all measures.

#### RFcluE versus RFclust

**Fig. 8:**
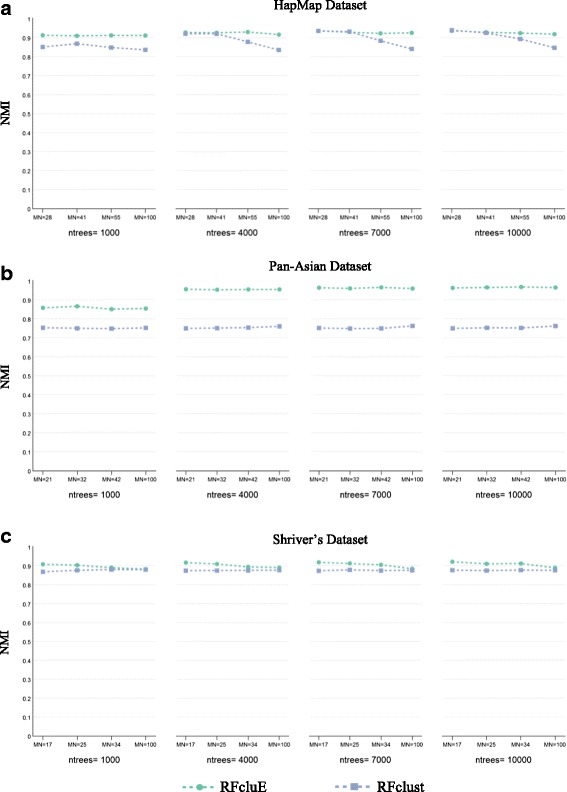
The impact of the change of RF parameters on the performance of RFclust vs. RFcluE. The figure shows the impact of the number of trees (*ntrees*) and the tree size controlled by the maximum number of leaf nodes (*MN*) om the performance of RFclust and RFcluE measured using NMI. **a** HapMap Dataset. **b** Pan-Asian Dataset. **c** Shriver’s Dataset

**Fig. 9:**
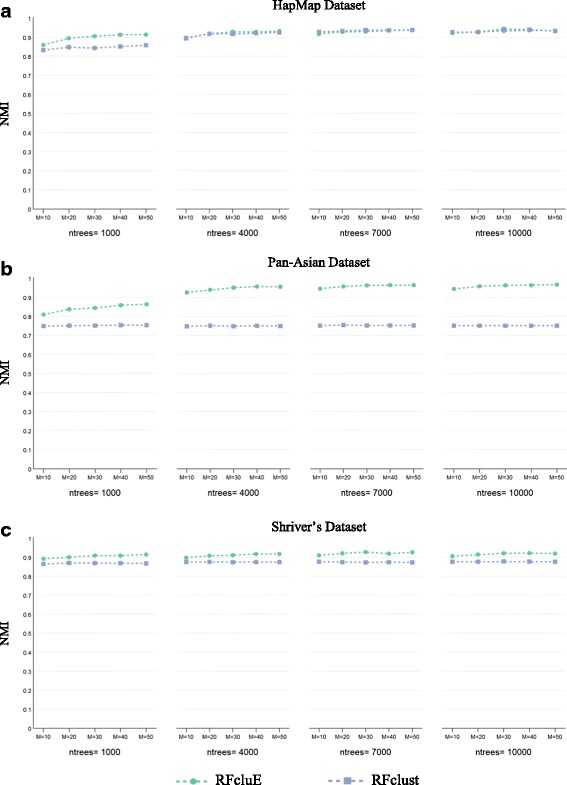
The impact of the change of the number of forests on the performance of RFclust vs. RFcluE. The figure shows the impact of the number of forests (*nforests*) on the performance of RFclust and RFcluE, which represents the ensemble size in RFcluE, measured using NMI across different numbers of trees (*ntrees*). **a** HapMap Dataset. **b** Pan-Asian Dataset. **c** Shriver’s Dataset

First, the effect of RF parameters was compared for both RFclust and RFcluE. As shown in Fig. 8, when RFclust is used, its performance is in most cases slightly changed as the tree size increases. An exception is HapMap, which shows a slight degradation in performance as the tree size increases. This confirms that, like RFcluE, building trees with $MN=\sqrt{N}$ is always sufficient for any dataset. On the other hand, RFclust performance was not affected by changing the number of trees per forest nor the number of forests, as shown in Fig. 9. However, its performance was slightly improved with HapMap when increasing the number of trees in the forest from 1000 to 4000, and was slightly improved thereafter. Overall, RFclust exhibited stable performance across different values of the number of trees per forest and the number of forests. In addition, small tree sizes are always efficient to provide robust results.

The plots in Fig. 8 show that when comparing the performance of RFcluE with that of RFclust under the same RF parameters, RFcluE performs significantly better for all parameter settings, especially for Pan-Asian datasets. Another parameter is the number of forests to be constructed, which represents the ensemble size within the RFcluE approach. Unlike RFclust performance, RFcluE performance was improved when increasing the number of forests (*M*), especially when using a smaller number of trees. As shown in Fig. 9, the performance of RFcluE was improved over that of RFclust as the number of trees was increased over different numbers of forests (*M*). One exception was that RFcluE performance for HapMap became similar to that of RFclust when increasing the number of trees from 1000 to 4000, and subsequently stabilized for both approaches. However, the performance of RFcluE was much better than that of RFclust over different numbers of trees for the Pan-Asian dataset. A similar observation can also be made for Shriver’s dataset, except that the performance of both approaches did not change much across different numbers of trees. This observation indicates that multiple forests with 1000 trees each were enough to discover the structure of Shriver’s dataset.

Overall, we can conclude that it is both crucial and more efficient to use RF clustering as a base clustering method within a cluster ensemble framework instead of averaging the proximities of several forests and then applying clustering. RFcluE exhibited better performance than RFclust, especially for clustering in the Pan-Asian dataset. One important observation was that the clustering performance of RFcluE was significantly improved by increasing the number of forests and the number of trees per forest; unlike RFclust, where these parameters became irrelative. Moreover, the performance of RFcluE was more robust than that of RFclust with respect to tree size as long as sufficient trees per forest were constructed.

### Diversity and quality analysis

The final experiment was conducted to assess the relationship between the diversity and quality of the generated ensemble and its influence on the quality of the ensemble’s final clustering. The diversity of base clusterings is a major factor that could affect the performance of the cluster ensemble approach. On the other hand, the evaluation of the quality of base clusterings is necessary to determine improvements in the quality of the final clustering of the cluster ensemble approach. To perform this experiment, the diversity and quality of base clusterings, as well as the quality of the ensemble’s final clustering, were calculated by applying Eq. (9), Eq. (10), and Eq. (8), respectively.

One source of diversity in base clustering is the number of clusters as an input to the base clustering method. *TrueK*, *FixedK*, and *RandomK* schemes, identified earlier, could generate different levels of diversity among base clusterings. Consequently, an experiment was conducted in order to study the diversity and quality of base clusterings generated by these different schemes with the following parameters: ($M=40,\kern0.5em ntrees=10000,\kern0.5em MN=\sqrt{N}$). Table 3 reports the diversity and quality of base clusterings, as well as the quality of the ensemble’s final clustering over the three datasets. Based on this table, we can observe that the *TrueK* scheme has the least diversity and the best quality of base clusterings; however, it produces the lowest quality of the ensemble’s final clustering. *FixedK* produces the highest quality of the final clustering among the three schemes for all datasets. This result confirms that selecting a greater number of clusters for base clustering methods than expected would introduce diversity within the ensemble. Thus, higher diversity could lead to more significant improvement in the quality of the ensemble’s final clustering. To this end, we can conclude that the quality of the base clusterings is not correlated with the quality of the cluster ensemble approach based on RFs, while combining base clusterings could produce a higher-quality final clustering result due to their diversity.

**Table 3:** The diversity and quality analysis of the three schemes

Dataset	Scheme	DS(P)	Q(P)	Q(P*)	Q(P*)-Q(P)
HapMap	FixedK	0.2697	0.7823	0.9353	0.1529
	RandomK	0.2614	0.7909	0.9208	0.1299
	TrueK	0.1542	0.8453	0.9057	0.0604
Pan-Asian	FixedK	0.2800	0.7794	0.9620	0.1826
	RandomK	0.3204	0.7727	0.9701	0.1974
	TrueK	0.1438	0.8799	0.9669	0.0870
Shriver’s	FixedK	0.1543	0.8592	0.9204	0.0612
	RandomK	0.2555	0.7680	0.8958	0.1279
	TrueK	0.1435	0.8530	0.8898	0.0368

The table shows the diversity and quality of the base clusterings (denoted by *DS (P)* and *Q (P)*, respectively) along with the quality of the ensemble’s final clustering, *Q* (*P* ^*^), for three datasets using the three different schemes: *FixedK*, *RandomK*, and *TrueK*

The other sources of diversity are bagging, random subspace, and synthetic data generation applied within unsupervised RF algorithms. Therefore, AWcluE and PCAcluE were developed as an ensemble version of the two single clustering methods, PCAclust and AWclust, to demonstrate how the diversity and quality of the base clustering method influence the performance of the entire ensemble, especially RF clustering as a base clustering method of RFcluE. Accordingly, PCAcluE and AWcluE are defined as ensemble-based clustering methods that apply different base clustering methods but utilize the same consensus function of RFcluE. On the one hand, PCAcluE applies PCA and then K-means as a base clustering method. On the other hand, AWcluE calculates ASD and then applies K-means as a base clustering method. For both methods, K-means with a random initialization is considered as a source of diversity that can produce different partitions of the data with varying accuracy.

Table 4 reports the results for each ensemble method over the three datasets using the same parameters (*M* = 40,$k=\sqrt{N}$). By comparing the diversity and quality between the three ensemble-based clustering methods, we can see that RFcluE has the most diverse ensemble across the three datasets with moderate quality. However, it achieves the best performance and exhibits greater improvements in the quality of the ensemble’s final clustering over that of base clusterings. From this experimental result, we conjecture that the RF clustering method is most beneficial when applied within a cluster ensemble framework. Computing the RF proximity enables viewing high-dimensional genetic data from different angles via bagging and random subspace, thus contributing to a more diverse ensemble than the two other ensemble-clustering methods. Thus, combining multiple RF clustering results using an ensemble approach produces better clustering result than a single RF clustering.

**Table 4:** The diversity and quality analysis of the three ensemble-based methods

Dataset	Method	DS(P)	Q(P)	Q(P*)	Q(P*)-Q(P)
HapMap	AWcluE	0.1912	0.8139	0.9148	0.1009
	PCAcluE	0.1429	0.7078	0.7510	0.0432
	RFcluE	0.2697	0.7823	0.9353	0.1529
Pan-Asian	AWcluE	0.1802	0.8356	0.9497	0.1141
	PCAcluE	0.0801	0.8427	0.8933	0.0506
	RFcluE	0.2800	0.7794	0.9620	0.1826
Shriver’s	AWcluE	0.1164	0.8804	0.8879	0.0074
	PCAcluE	0.0896	0.8236	0.8351	0.0115
	RFcluE	0.1543	0.8592	0.9204	0.0612

The table shows the diversity and quality of the base clusterings (denoted by *DS* (*P*) and *Q* (*P*), respectively) along with the quality of the ensemble’s final clustering, *Q* (*P* ^∗^), for three datasets using the three ensemble-based clustering methods: PCAcluE, AWcluE, and RFcluE

## Conclusions

This paper has presented RFcluE, a cluster ensemble approach based on an RF algorithm, to address the problem of population structure analysis. This approach is composed of two stages: ensemble construction, in which an RF-based clustering method is applied to generate a set of clusterings for the same dataset; and consensus function, which integrates all the clusterings to produce a final data clustering. Many experiments were conducted to empirically investigate the potential of the RFcluE approach on real genetic datasets in order to uncover the substructure of populations. In addition, a comparison study was carried out to compare RFcluE performance against existing, popular clustering methods for population structure analysis. The experimental results illustrated that the proposed approach, RFcluE, outperformed the other clustering approaches, providing more accurate results. Moreover, the experimental results indicated that combining multiple clusterings, generated based on RFs, within a cluster ensemble produces high quality and robust clustering results in comparison to a single run of RF clustering. This improvement in performance is a consequence of feeding the ensemble with diverse views of high-dimensional genetic data obtained through bagging and random subspace, the two key features of the RF algorithm. To conclude, the major contributions of this paper are proposing and evaluating a cluster ensemble approach based on RFs and demonstrating its effectiveness for high-dimensional, real genetic data. The paper also illustrated that applying a cluster ensemble approach to combine multiple RF clusterings produces more robust and high-quality clustering results than clustering based on averaging the proximities derived from multiple forests. Future work should include the application of the RFcluE approach to other high-dimensional biological data.

## Abbreviations

AC: Accuracy
ARI: Adjusted Rand index
ASD: Allele-sharing distance
ASRS: Approximate SimRank-based similarity
CO: Co-association matrix
CTS: Connected-triple-based similarity
LD: Linkage disequilibrium
MDS: Multidimensional scaling
NMI: Normalized mutual information
PCA: Principal component analysis
RFcluE: Random Forest cluster Ensemble
RFclust: Random Forest clustering
SNPs: Single nucleotide polymorphisms
SRS: SimRank-based similarity
